# Robust Image Population Based Stain Color Normalization: How Many Reference Slides Are Enough?

**DOI:** 10.1109/OJEMB.2023.3234443

**Published:** 2023-01-05

**Authors:** Jose L. Agraz, Caleb M. Grenko, Andrew A. Chen, Angela N. Viaene, MacLean D. Nasrallah, Sarthak Pati, Tahsin Kurc, Joel Saltz, Michael D. Feldman, Hamed Akbari, Parth Sharma, Russell T. Shinohara, Spyridon Bakas

**Affiliations:** Center for Biomedical Image Computing and Analytics (CBICA) Philaldelphia PA 19139 USA; Department of Pathology and Laboratory Medicine, Perelman School of Medicine 14640 Philaldelphia PA 19139 USA; Department of Radiology at Perelman School of MedicineUniversity of Pennsylvania 6572 Philaldelphia PA 19139 USA; Department of Pathology and Laboratory Medicine, Perelman School of MedicineUniversity of Pennsylvania and the Center for Interdisciplinary Studies 6572 Davidson College NC 28035 USA; Penn Statistical Imaging and Visualization Endeavor (PennSIVE)University of Pennsylvania 6572 Philaldelphia PA 19139 USA; Department of Pathology and Laboratory Medicine, Children's Hospital of PhiladelphiaUniversity of Pennsylvania 6572 Philaldelphia PA 19139 USA; Department of Pathology and Laboratory Medicine, Perelman School of MedicineUniversity of Pennsylvania 6572 Philaldelphia PA 19139 USA; CBICA and Department of Pathology and Laboratory Medicine, Perelman School of MedicineUniversity of Pennsylvania 6572 Philaldelphia PA 19139 USA; Department of Radiology at Perelman School of MedicineUniversity of Pennsylvania 6572 Philaldelphia PA 19139 USA; Department of Biomedical InformaticsStony Brook University 12301 Stony Brook NY 11794-0751 USA; CBICA and the Department of Radiology, Perelman School of MedicineUniversity of Pennsylvania 6572 Philaldelphia PA 19139 USA; Symphony Health 348949 Philaldelphia PA 19139 USA; CBICA and the Penn Statistical Imaging and Visualization Endeavor (PennSIVE)University of Pennsylvania 6572 Philaldelphia PA 19139 USA; CBICA, and the Department of Pathology and Laboratory Medicine, Perelman School of MedicineUniversity of Pennsylvania 6572 Philaldelphia PA 19139 USA; Department of Radiology, Perelman School of MedicineUniversity of Pennsylvania 6572 Philaldelphia PA 19139 USA

**Keywords:** Power law, Pareto principle rule, Stain normalization bias problem, Ivy GAP, Whole slide image

## Abstract

Histopathologic evaluation of Hematoxylin & Eosin (H&E) stained slides is essential for disease diagnosis, revealing tissue morphology, structure, and cellular composition. Variations in staining protocols and equipment result in images with color nonconformity. Although pathologists compensate for color variations, these disparities introduce inaccuracies in computational whole slide image (WSI) analysis, accentuating data domain shift and degrading generalization. Current state-of-the-art normalization methods employ a single WSI as reference, but selecting a single WSI representative of a complete WSI-cohort is infeasible, inadvertently introducing normalization bias. We seek the optimal number of slides to construct a more representative reference based on composite/aggregate of multiple H&E density histograms and stain-vectors, obtained from a randomly selected WSI population (WSI-Cohort-Subset). We utilized 1,864 IvyGAP WSIs as a WSI-cohort, and built 200 WSI-Cohort-Subsets varying in size (from 1 to 200 WSI-pairs) using randomly selected WSIs. The WSI-pairs' mean Wasserstein Distances and WSI-Cohort-Subsets' standard deviations were calculated. The Pareto Principle defined the optimal WSI-Cohort-Subset size. The WSI-cohort underwent structure-preserving color normalization using the optimal WSI-Cohort-Subset histogram and stain-vector aggregates. Numerous normalization permutations support WSI-Cohort-Subset aggregates as representative of a WSI-cohort through WSI-cohort CIELAB color space swift convergence, as a result of the law of large numbers and shown as a power law distribution. We show normalization at the optimal (Pareto Principle) WSI-Cohort-Subset size and corresponding CIELAB convergence: a) Quantitatively, using 500 WSI-cohorts; b) Quantitatively, using 8,100 WSI-regions; c) Qualitatively, using 30 cellular tumor normalization permutations. Aggregate-based stain normalization may contribute in increasing computational pathology robustness, reproducibility, and integrity.

## I. Introduction

There is a perennial interest in digital pathology whole slide image (WSI) stain color normalization, with regards to personalized medicine in automated disease diagnosis and patient management [1], [2], [3], [4], [5], [6], [7], [8]. State-of-the-art stain separation normalization methods use a single WSI as the reference standard for normalization of a WSI-cohort [2], [9], [10], [11], [12]. However, using a single WSI as a reference resembling an entire WSI-cohort is infeasible, as WSI regions of interest (ROI) are morphologically unique, and hence introducing an unintended color bias, resulting in inferior algorithm generalizability [13], [14], [15], [16], [17]. Often, multiple WSI are selected, the mean stain segmentation accuracy recorded, and the highest scoring images used as reference for WSI-cohort color normalization [18]. Alternatively, a random WSI is selected as reference for WSI-cohort color normalization potentiating resulting in worse color bias. Both WSI selection methods are impractical, error prone, and do not completely address the color normalization bias problem. Most importantly, these WSI selection approaches deteriorate neural network model generalization, while magnifying data domain shift, making unseen tumor data classification significantly challenging [19], [20], [21], [22], [23], [24].

We discuss a population (WSI-cohort subset) based hematoxylin and eosin (H&E) WSI-Cohort stain color normalization, not in terms of a single WSI, as a stain color normalization reference standard, but by considering the WSI-cohort subset H&E density histograms and Stain Vectors aggregates. The WSI-cohort subset size where stain CIELAB color space (also referred to as Lab) intensity channel (LabIC) [25] converges and beyond are representative of the entire WSI-cohort and the WSI-cohort subset aggregate suitable as a color normalization reference standard. We build upon an initial study of WSI color convergence tasked for a smaller Ivy GAP [26], [27], [28] WSI-cohort (n = 509) [29]. The present work enhances the initial approach using a larger Ivy GAP WSI-cohort (n = 1864), demonstrating LabIC convergence and developing an optimization method based on the Pareto principle 80/20 rule [30], [31], [32], [33]. The WSI-cohort LabIC convergence curve follows the law of large numbers [34], [35], [36], shown as the Wasserstein distance standard deviation between random WSI pairs, and is described by a right long-tailed power law distribution [32].

There is an extraordinary amount of research in stain color normalization [37], [38], [39] falling into two categories; color deconvolution and statistical pixel clustering. The color deconvolution method separates stains, normalizing stains individually, and rejoining stains into a single image. However, the method assumes accurate stain separation limited by a tissue structure spatial dependency. From creation, color deconvolution has evolved from a high-performance stain normalization requiring prior reference WSI stain vectors [40], to more accurate methods based on deconvolution in the optical density [11] and in CIELAB color spaces with a lesser tissue structure spatial dependency [12].

In addition to the law of large numbers, power law, and Pareto principle theory, we use high performance computing (HPC) for the analysis of large amounts of WSIs (i.e., Big Data) through stain deconvolution, histogram matching, and probability distributions distance measurements. Finally, we present color intensity convergence through WSI, ROIs, and large patch normalization permutations, as well as stain color normalization based on an optimal WSI-cohort subset size at the pareto principle 80/20 rule. The above method may theoretically be used for stains, diseases, and normalization methods other than H&E, glioblastoma, and stain deconvolution method. In these new conditions the key question is how quickly stains color intensity convergence reaches an asymptote.

## II. Materials and Methods

**Fig. 1.**
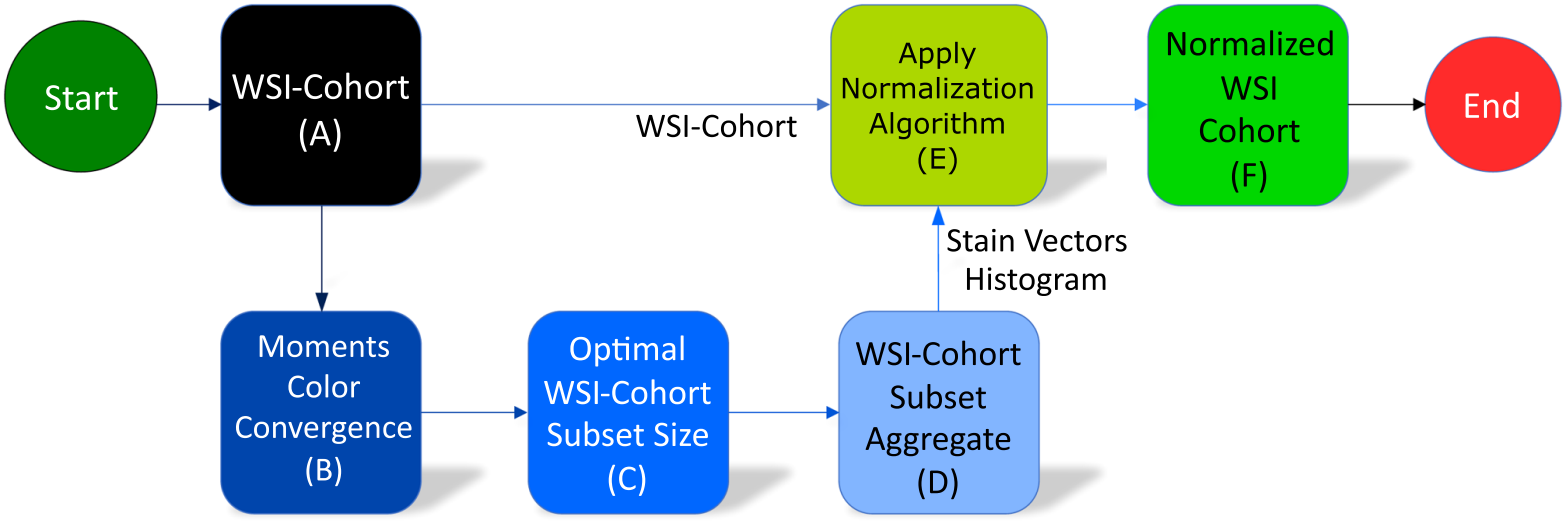
Ivy GAP Cohort Color Normalization Work Flow Analysis. (A) Input: WSI-cohort, (B) Moments LabIC convergence approximation, (C) Optimization using the Pareto principle 80/20 rule, WSI-cohort subset size of randomly selected WSI's, (D) WSI-cohort subset histogram and stain vectors aggregates, (E) WSI-cohort color normalization by reference histogram and stain vectors transfer, (E) Output: Normalized Ivy GAP WSI-cohort.

The proposed method aims to find the optimal WSI-cohort subset size representative of a complete WSI-cohort using the pareto principle 80/20 rule. The resulting optimal WSI-cohort subset aggregate (histogram and stain vectors) used as a normalization reference standard, when normalizing a WSI-cohort using the structure-preserving color normalization algorithm [12] (Fig. 1).

### A. WSI-Cohort Subset Selection

**Fig. 2.**
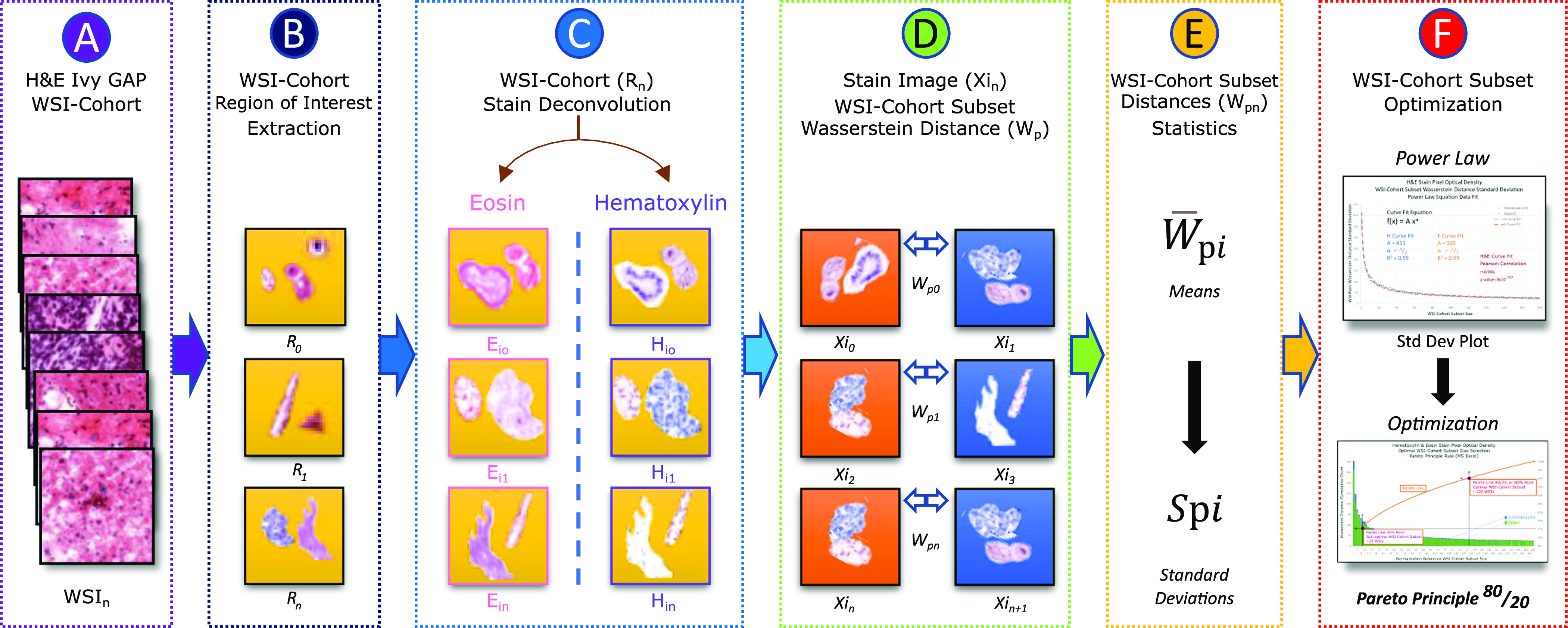
Optimal WSI-cohort subset size process. (A) Ivy GAP 1864 WSI-cohort. The WSI-cohort was validated by discarding WSIs with markings and abnormal discoloration. (B) Background isolated ROIs. (C) Deconvolution separated H&E stains. (D) 200 WSI-cohort subsets creation. (E) WSI-pairs Wasserstein Distance means and WSI-cohort subset standard deviations calculation. Repeated for 1000 mean data points per WSI-cohort subset. (F) Pareto Principle approximation.

A WSI-cohort of 1864 Ivy GAP [26], [27], [28] WSIs was selected (Fig. 2(A)), the background removed (Fig. 2(B)), and stain deconvolution separated in H&E WSI-cohorts [9], [10], [11], [12], [16] (Fig. 2(C)). Then, 200 new WSI-cohort subsets created using randomly selected WSI-pairs with WSI replacement. These subsets varied in size from 1 to 200 WSI-pairs and the mean Wasserstein Distance ($W_ {p}$) calculated (Fig. 2(D)). The process was repeated for the 200 WSI-cohort subsets and stains, for a total of 1,000 permutations per WSI-cohort subset and respective standard deviations ($S_ {pi}$) calculated (Fig. 2(E)). Finally, the stains standard deviations plotted, verifying $S_ {pi}$ power law behavior. The optimal cohort WSI subset size was calculated at the Pareto principle 80/20 rule [30] (Fig. 2(F)) and the Pearson correlation Test applied to H&E stain power law curves for curve similarity quantification.

### B. Wasserstein Distance for Normalization Evaluation

We compute the Wasserstein distance for each WSI-pair across two different cohorts of equal size. In computing the average Wasserstein Distance across increasingly large cohorts, we show that results follow the law of large numbers, as results converge to a stable value. In general, the law of large numbers states that the sample mean converges to the true average value, as the number of samples increases. Here, samples consist of pairwise Wasserstein distances between individual WSIs. The mean of pairwise distances is often called the Gini's coefficient and is used as a standard measure of spread [46], [47], [48]. Let $D$ be a random variable representing the Wasserstein distance between a pair of WSI from separate cohorts with true expected value $E(D)=\mu$. For independent and identically distributed samples from $D$, the strong law of large numbers states that the sample average of Wasserstein distances will converge almost surely to the expected value [49]. That is, as the sample size $n$ goes to infinity, the sample average of Wasserstein distances $\overline{D_{n}}$ follows:

\begin{equation*}
\overline{D_{n}} \xrightarrow []{a.s.} \mu \tag{1}
\end{equation*}

### C. Optimal WSI-Cohort Subset Size and the Pareto Principle

At larger WSI-cohort sizes, the effectiveness of normalization is reflected in the decreasing spread of Wasserstein distances measured across WSI-pairs. We hypothesize that the WSI-cohort Wasserstein distance standard deviation may be described by a power law

\begin{equation*}
p(x)=Ax^{-\alpha } \tag{2}
\end{equation*}

where $p(x)$ is the distribution, A is the asymptote, and $\alpha$ is the data estimated exponent. This equation has been used as a model for many phenomena in nature and society [41].

We select an optimal cohort size as one that provides sufficient reduction of Wasserstein distance standard deviation. We adapt the widely used in finance Pareto analysis technique [33] available as a function in Microsoft Excel. Following Pareto analysis, we choose an optimal cohort size that reduces the standard deviation by 80% of the maximum reduction, measured at the largest cohort sizes of 200 WSIs for H&E stains. This optimal cohort size provides sufficient stability in the pairwise distances between WSI, reflecting controlled differences between normalized WSIs [32].

### D. Optimal WSI-Cohort Subset Normalization Parameters

After WSI-cohort stain separation and the optimal WSI-cohort subset size ($S_{n}$) are found, a $S_{n}$ of random WSI were selected and labeled as the optimal WSI-cohort subset. Then, the optimal WSI-cohort subset $\hat{W}$ (dictionary learning) and $\hat{H}$ (sparse coding) matrices were calculated [12]. Finally, the optimal WSI-cohort subset $W_{op}$ and $H_{op}$ calculated by

\begin{align*}
W_{op} & =\frac{1}{S_{n}}\sum _{k=1}^{S_{n}} W_{k} \tag{3}
\\
H_{op} & =\frac{1}{S_{n}}\sum _{k=1}^{S_{n}} H_{k} \tag{4}
\\
H_{k} & =\frac{H_{i}-H_{\min }}{H_{\max }-H_{\min }} \ast C \tag{5}
\end{align*}

Where the histogram ${H}$ bin size is taken from the image with the largest Knuth optimal bin size [51] and the resulting bin size increased to next power of 2 for hardware efficiency. Then, the histogram normalized to a constant $C$. Lastly, image normalization is performed through $H_{op}$ and $W_{op}$ matching

### E. Quantitative WSI-Cohort LabIC Convergence

First, the WSI-cohort subset aggregated stain vectors and histogram were calculated, followed by complete 1864 WSI-cohort normalization, WSIs transformation to CIELAB color space, and respective intensity channel extraction. Then, the intensity channel means per WSI and standard deviation per WSI-cohort were calculated. The process was repeated for 50 permutations and five WSI-cohort subset sizes (1, 10, 120, 1000, and 1864 WSIs), resulting in an analysis of 500,000 WSIs. Finally, the Levene's test (Levene, 1960) was applied between adjacent WSI-cohort subset-based normalizations and range measured for variance quantification across WSI-cohort subsets.

### F. Quantitative ROI LabIC Convergence

First, the WSI-cohort subset aggregated stain vectors and histogram were calculated, followed by Ivy GAP WSI file names 300933007 and 101713101 normalization, WSIs transformation to CIELAB color space, and respective LabIC extraction. Subsequently, the LabIC means per WSI and standard deviation per cohort calculated. The process was repeated for 225 permutations and five WSI-cohort subset sizes (1, 10, 100, 1000, and 1864 WSIs), and nine WSI annotations or ROIs (Leading Edge, Infiltrating Tumor, Cellular Tumor, Tumor Perinecrotic Zone, Psuedopalisading Cells around Necrosis, Tumor Microvascular Proliferation, Hyperplastic Blood, Necrosis, and Pseudopalisading Cells with no visible Necrosis), resulting in an analysis of 10,000 ROIs. Finally, the Levene's test was applied between adjacent WSI-cohort subset-based normalizations and range measured for variance quantification across WSI-cohort subsets.

### G. Qualitative Large Patch LabIC Convergence

In addition to quantitative convergence representation, we show a qualitative representation of convergence using different WSI-cohort subset sizes. First, the WSI-cohort subset aggregated stain vectors and histogram were calculated, followed by Ivy GAP WSI file name 300933007 normalization and 5,000 by 5,000-pixel Cellular Tumor ROI patch extraction (x = 4300, y = 6750), corresponding LabIC extracted, and shown as a heatmap. The process was repeated for six permutations and five cohort subset sizes used as normalization reference (1, 10, 100, 1000, and 1800 WSIs).

## III. Results

### A. WSI-Cohort Color Moments Convergence

**Fig. 3.**
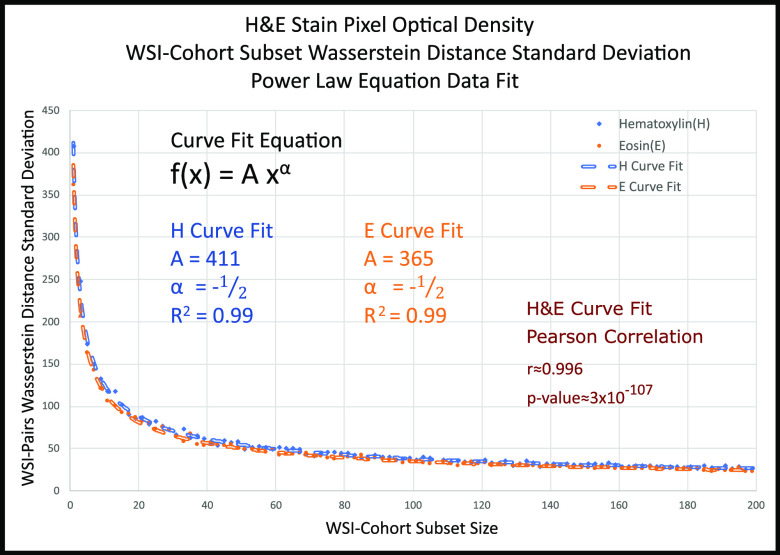
H&E pixel optical density Wasserstein distance standard deviation curves. Curves show the convergence (decay) in Wasserstein distance standard deviation between WSI-pairs. H&E power law curves (2) are of similar shape with parameters: A = 411 and A = 365 for Hematoxylin and Eosin respectively, with $\alpha =-\frac{1}{2}$ and $R^{2}=0.99$, and Pearson correlation $r=0.996$ with a p-value$< 0.05$.

**Fig. 4.**
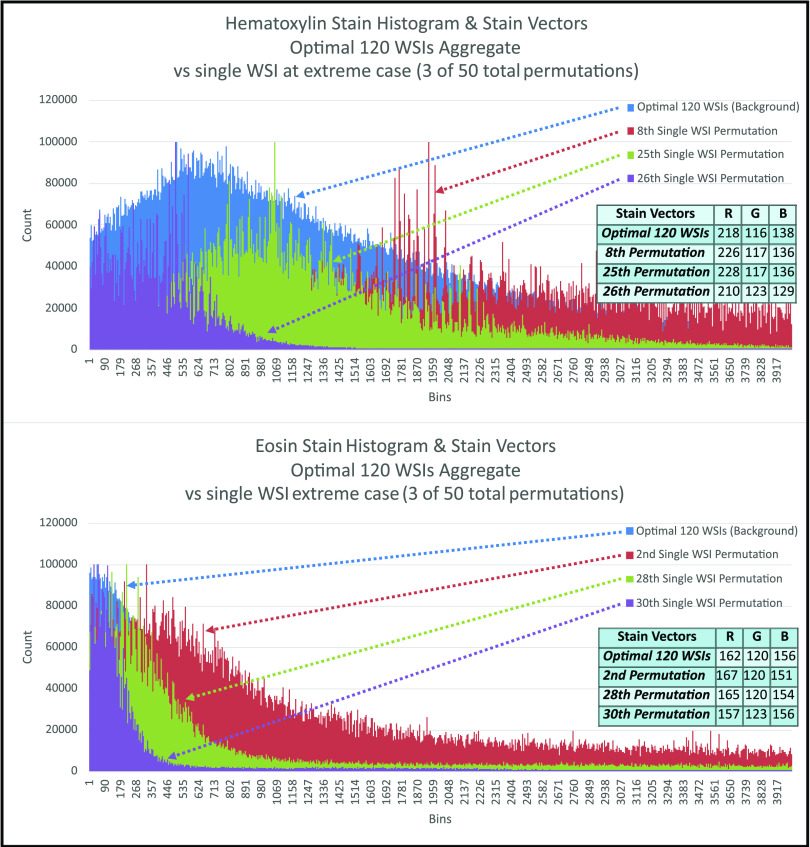
H&E histograms and corresponding stain vectors, at 120 WSI-Cohort subset optimal size and three permutations of single WSI at extreme cases for comparison. Top:Hematoxylin and Bottom: Eosin.

The H&E stain color WSI-pairs Wasserstein distance standard deviation are shown as a power law curves (2) and curve fitting simulations found to achieve $A=411$ and $A=365$, for the Hematoxylin and Eosin stains respectively, with an $\alpha =-\frac{1}{2}$ and $R^{2}=0.99$, where $R^{2}$ is the multiple correlation coefficient. Furthermore, H&E stain curves Pearson correlation Test, two-tailed distribution, shows $r = 0.996$ with a p-values = $3 \times 10^{-107}$ (Fig. 3). Lastly, the optimal 120 WSI-Cohort subset aggregated histogram and three permutations of single WSI histograms at extreme cases for Hematoxylin ($8{\text{th}}$, $25{\text{th}}$, and $26{\text{th}}$ permutation) and Eosin stains ($2{\text{th}},28{\text{th}},30{\text{th}}$ permutation) are shown along with corresponding stain vectors are shown (Fig. 4) [1].

### B. Quantitative WSI-Cohort Normalization Convergence

**Fig. 5.**
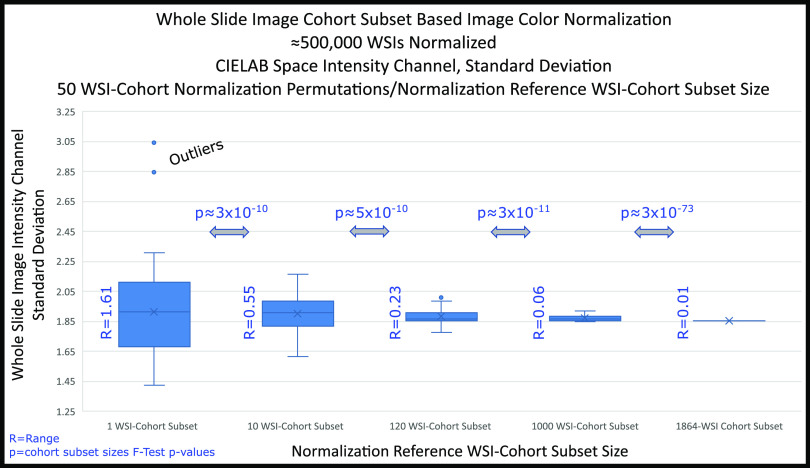
LabIC convergence analysis for various WSI-cohort subsets. Nearly 500,000 WSIs processed in a HPC environment for two months. Applied Levene's test to adjacent WSI-cohort subsets. p-values $< 0.05$ show a statistical difference while decreasing ranges show convergence (exponential decay).

The color normalization for the complete WSI-cohort using five WSI-cohort subset sizes (1, 10, 100, 1000, and 1864 WSIs), as normalization references, yielded Levene's test p-values $< 0.5$ and decreasing range values results (Fig. 5), and values shown in Supplementary Information (SI), Tables I and II respectively.

### C. Quantitative WSI-Cohort ROI Normalization Convergence

**Fig. 6.**
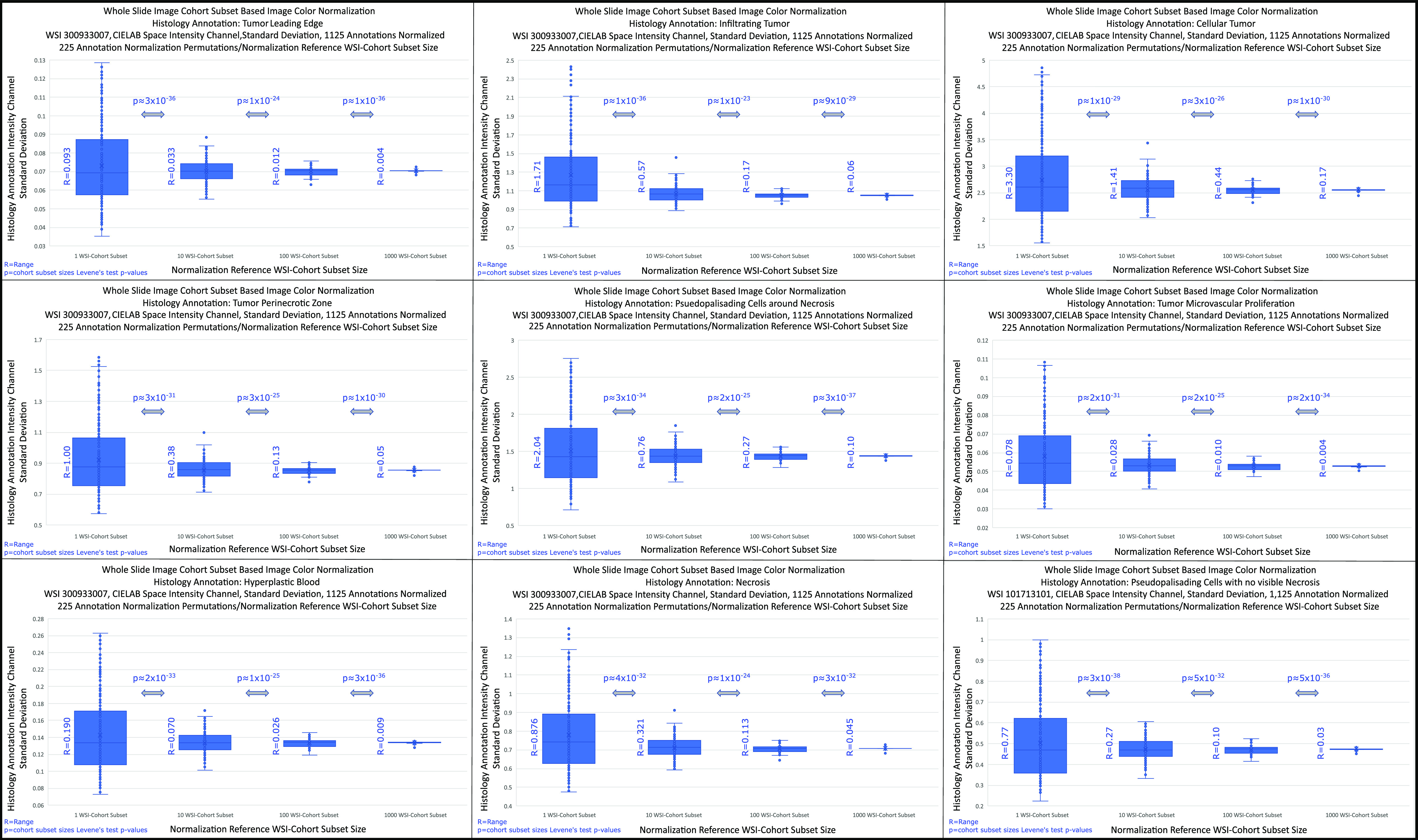
Nine glioblastoma ROIs LabIC convergence. ROIs were normalized using four WSI-cohort subset sizes (1,10,100, and 1000 WSIs), as normalization reference. Applied Levene's Test to adjacent WSI-cohort subsets, p-values and ranges shown.

Quantitative normalization LabIC convergence for nine ROIs using four WSI-cohort subsets sizes (1,10,100,1000 WSIs), as normalization references, yielded Levene's test p-values $< 0.5$ and decreasing ranges results (Fig. 6), values shown in SI, Tables III and IV respectively.

### D. Qualitative Large Patch Convergence

**Fig. 7.**
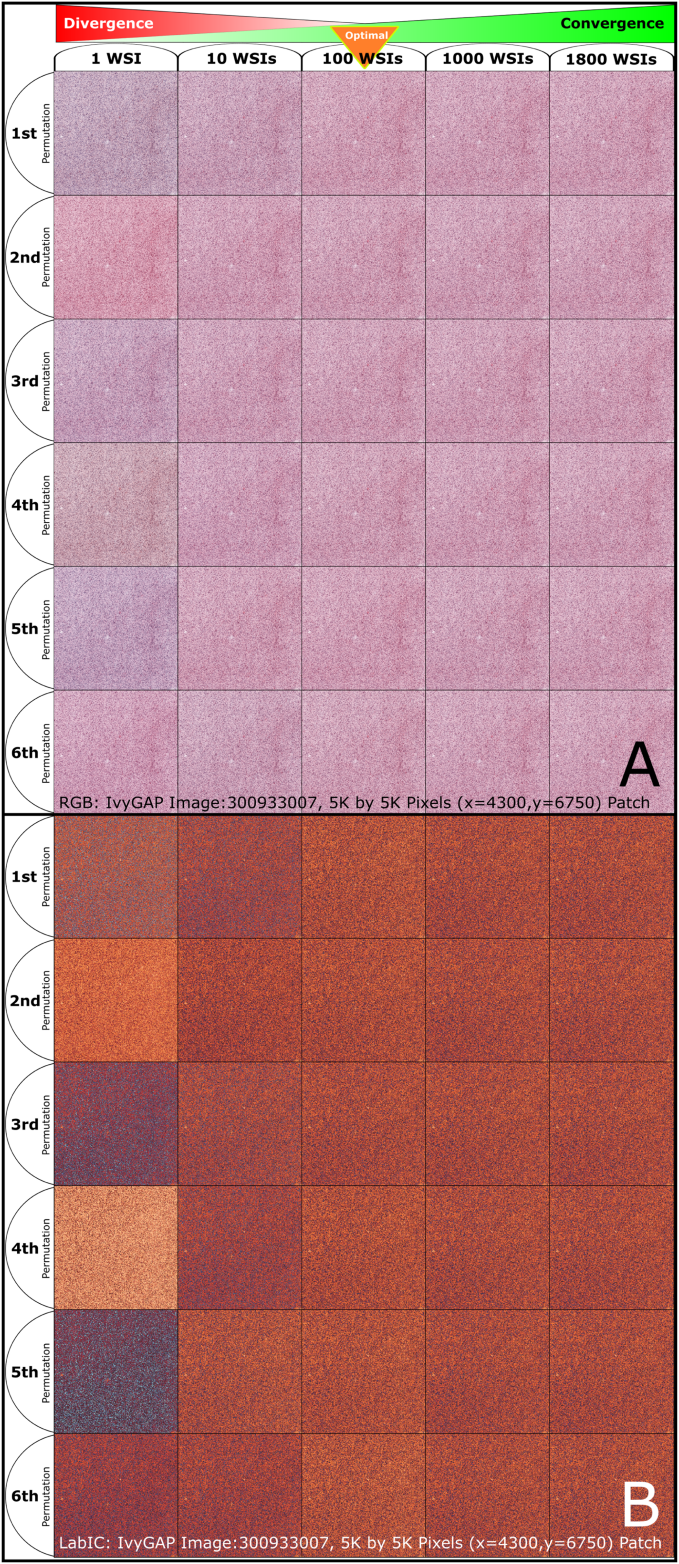
Qualitative Cellular Tumor ROI large patch normalization convergence. (A) RGB, large patch normalization convergence increases as the number of WSIs increase in a WSI-cohort subset used as a normalization reference. (B) LabIC, clearer representation of large patch normalization convergence. Large patch normalized using five WSI-cohort subsets sizes (1, 10, 100, 1000, and 1864 WSIs), as a normalization reference shown for six permutations using WSI(s) patches with the most extreme results.

Qualitative representation of Cellular Tumor ROI large patch normalization for RGB and LabIC convergence using five WSI-cohort subset sizes (1, 10, 100, 1000, 1800 WSIs), as normalization references, are shown (Fig. 7). RGB and LabIC patches become more homogeneous as the size of the normalization reference WSI-cohort subset increases

### E. Pareto Principle 80/20 Rule Normalization

**Fig. 8.**
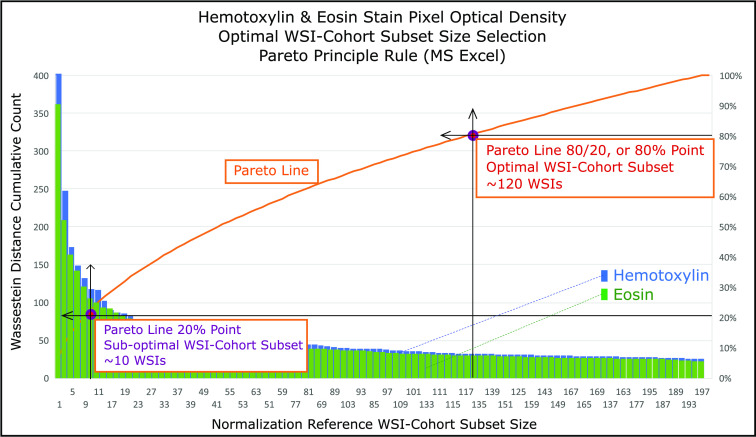
Pareto Principle determination of sub-optimal and optimal H&E WSI-cohort sizes using Wasserstein distances standard deviations.

The Pareto Principle 80/20 rule was applied to the H&E stains power law curves (Fig. 3), yielding optimal WSI-cohort subset size at the 80% point, or 120 WSIs (Fig. 8). For comparison, antithetical points were selected at a) single WSI, b) sub-optimal WSI-cohort subset size at 20%, or 10 WSIs and c) Full WSI-Cohort or 1864 WSIs. Converging Wasserstein distance standard deviation values shown in SI, Table V.

## IV. Discussion

Histological WSI often show color nonconformity caused by materials, equipment, and staining protocols differences. Although, pathologists compensate for these irregularities, these inaccuracies hinder automated computational analysis by accentuating data domain shift and algorithm generalizability that aid the diagnoses and treatment of disease.

We have shown the H&E density histogram and stain vectors composite or aggregates of a number of randomly selected WSI population (WSI-cohort subset) within a WSI-cohort, can be used as the normalization reference standard, since the WSI-cohort LabIC convergence follows the law of large numbers theorem and power law distribution. The WSI-cohort subset's stain vectors and histogram aggregates are representative of a given WSI-cohort with greater fidelity than standard approaches using a single WSI normalization reference. This new approach yields effective Ivy GAP 1864 WSI-cohort normalization results without color nonconformity and the unintended color bias from single WSI color normalization.

Using the Wasserstein distances means, we have calculated the cohort subsets' standard deviation curves. These curves are shown as a power law (Pareto Distribution), for both H&E stains (2). Worth noticing, a single curve is needed to show power law trend, as both H&E power curves are statistically identical, sharing the same decay ($\alpha$), and a strong Person correlation (Figs. 3 & 8). Furthermore, standard deviations distance curves beyond the shown image-pairs yielded negligible distances contributions (not shown).

Quantitative, WSI-cohort normalization LabIC convergence is shown two-fold through Wasserstein Distance standard deviation values in the LabIC: a) at the complete WSI and b) WSI ROI levels. More specifically, the standard deviations quantification was performed at four WSI-cohort subsets (1, 10, 100, and 1000 WSIs) with 50 permutations/cohort-subset and five WSI-cohort subsets (1, 10, 120, 1000, 1864 WSIs) with 225 permutations/cohort-subset, for the complete WSI and nine WSI ROI respectively. All standard deviations show LabIC convergent behavior with Levene's test p-values $< 0.05$, and decaying ranges, as WSI-cohort subsets size increase (Figs. 5 & 6). In addition, we have shown qualitative normalization convergence validation at the patch level by observing a normalized cancer tumor ROI patch (5,000 by 5,000-pixels) in RGB space and LabIC heatmaps at four WSI-cohort subsets (1, 10, 100, 1000, 1800 WSIs) for six permutations (Fig. 7)

Moreover, we have determined the optimal cohort subset size, as the normalization reference standard, by utilizing the Pareto principle. The Pareto principle 80/20 rule states 80% of the effects are the result of 20% of the causes. For normalization convergence comparison, we normalized the Ivy GAP 1864 WSI-cohort utilizing the followings as a normalization reference standard: a) a single WSIs, b) sub-optimal WSI-cohort subset (10 WSIs), c) optimal WSI-cohort subset (120 WSIs), and d) full WSI-cohort (1864 WSIs), and show Levene's test p-values and ranges. The sub-optimal and optimal cohort subsets were found by applying the Pareto Principle at 20% and 80/20 rule respectively, where variances converge at optimal cohort subset size through full cohort. These results demonstrate 120 random WSIs as optimal WSI-cohort subset size as a normalization reference (Fig. 8).

The determination of 120 random WSIs, as the optimal cohort subset size normalization reference, instead of single WSI, suggests a new robust approach in image pre-processing for network model development, as well as a more accurate clinical evaluation. Furthermore, our analysis shows no statistical difference in the Wasserstein distance standard deviations between H&E stains (Fig. 3). Thus, using a single stain (H or E) analysis could yield faster results than utilizing both H&E datasets.

The method is limited by the large amount of HPC resources required for computations and aggravated by the large size of the images in the Ivy-GAP WSI-cohort (1864 images). The following calculations show the longest computation effort by order of difficulty: a) the asymptote for the Wasserstein Distance [44] standard deviations between WSI-pairs (Fig. 3), b) the optimal histogram bin size [51] for the WSI-cohort, and c) demonstrating quantitative normalization convergence in WSIs (Fig. 5) and RIOs (Fig. 6) in multiple WSI-cohort subsets. Processing the 500,000 WSIs used in calculations required 75% of CBICA's HPC for over a two-month period. Future explorations in python-dask parallel-computing and sophisticated color convergence optimization algorithms will be particularly rewarding.

## V. Conclusion

This paper presents a technique for employing a 120 WSIs as the color normalization reference standard for structure-preserving color normalization of a given WSI-cohort. The technique demonstrates that a WSI-cohort subset stain vectors and histogram aggregates are representative of a given WSI-cohort with greater fidelity than a single WSI. Furthermore, the use of either H or E Wasserstein distance standard deviations is all that is required to deduce the optimal WSI-cohort subset of 120 WSIs for WSI-cohort normalization.

The analysis shows that the common practice of using a single WSI, as reference standard, results in a significant skewed WSI-cohort normalization. However, utilizing an optimal WSI population (WSI-cohort subset) size of 120 WSIs (Pareto principle 80/20 rule), as the reference standard, results in an accurate WSI-cohort color normalization without the inherent color normalization bias.

Theoretically, this approach could minimize discrepancies in glioblastoma histological evaluations. Future implementations utilizing a fully parallel-based-computation color convergence approach and improved stain separation methods could also result in a more efficient analysis.

## Supplementary Materials

We provide quantitative data in table form showing LabIC convergence at two levels: WSI-cohort and individual WSI-cohort ROIs. In addition, included are: a) video, still, and Power Point presentation Graphical abstracts and b) Python Code Ocean capsule to evaluate results with sample images, test cases, and a Power Point presentation describing Python code functions. The Code Ocean python capsule is available at https://codeocean.com/capsule/3803185. Note: Code Ocean is a cloud-based computational reproducibility platform fully integrated with IEEE Xplore.
